# Robust Weighted *l*_1,2_ Norm Filtering in Passive Radar Systems

**DOI:** 10.3390/s20113270

**Published:** 2020-06-08

**Authors:** Baris Satar, Gokhan Soysal, Xue Jiang, Murat Efe, Thiagalingam Kirubarajan

**Affiliations:** 1 Department of Electrical and Electronics Engineering, Ankara University, Golbasi, Ankara 06830, Turkey; bsatar@ankara.edu.tr (B.S.); efe@eng.ankara.edu.tr (M.E.); 2 School of Electronic Information and Electrical Engineering, Shanghai Jiao Tong University, Shanghai 200240, China; xuejiang@sjtu.edu.cn; 3 Department of Electrical and Computer Engineering, McMaster University, Hamilton, ON L8S 4L8, Canada; kiruba@mcmaster.ca

**Keywords:** delay-Doppler estimation, target detection, impulsive noise, passive radar, *l_p_* norm

## Abstract

Conventional methods such as matched filtering, fractional lower order statistics cross ambiguity function, and recent methods such as compressed sensing and track-before-detect are used for target detection by passive radars. Target detection using these algorithms usually assumes that the background noise is Gaussian. However, non-Gaussian impulsive noise is inherent in real world radar problems. In this paper, a new optimization based algorithm that uses weighted $l_{1}$ and $l_{2}$ norms is proposed as an alternative to the existing algorithms whose performance degrades in the presence of impulsive noise. To determine the weights of these norms, the parameter that quantifies the impulsiveness level of the noise is estimated. In the proposed algorithm, the aim is to increase the target detection performance of a universal mobile telecommunication system (UMTS) based passive radars by facilitating higher resolution with better suppression of the sidelobes in both range and Doppler. The results obtained from both simulated data with α stable distribution, and real data recorded by a UMTS based passive radar platform are presented to demonstrate the superiority of the proposed algorithm. The results show that the proposed algorithm provides more robust and accurate detection performance for noise models with different impulsiveness levels compared to the conventional methods.

## 1. Introduction

Passive radar systems are radar systems capable of target detection with one or more receivers without having any active transmitter. Passive radar systems are non-cooperative systems in that these can detect targets using illuminators of opportunity that are already in the environment [1]. Detection is carried out using different waveform types, such as television, radio, and mobile communication signals. Some of the benefits of passive radar systems include resilience to jamming and elimination of the need for frequency allocation [1]. Furthermore, the ubiquity of communication signals in the environment enables passive radar to be useful in scenarios where active radars are not available or inhibited.

One of the most commonly used methods for target detection is the conventional matched filter [2]. The operation of this method can be summarized as the estimation of the range and the Doppler of the target by comparing the noisy reference signal with the signal reflected from a target through a matched filter. There are many studies in the literature on the detection of low observable targets with the help of passive radar systems using the conventional matched filter. In [3], digital video broadcast (DVB) signals were collected for detection micro-Doppler signature. Then, normalized least mean squares (NLMS)-based algorithm was applied to suppress the interference, and a range-Doppler map was produced by calculating the cross-ambiguity function of the signals. In [4], two double-row Yaggi antennas was used with digital televison based passive radar systems. The challenge of low-observable target detection in the presence of sea clutters was tackled by using multi-channel adaptive filters. Pulse compression technique was used to create a range-Doppler map. In [5], the range resolution was improved by unmatched filtering to detect closely moving targets, and it was shown that the matched and unmatched filtering performances were almost the same for targets moving at different speeds. In [6], an unmanned aerial vehicle (UAV) was detected among many flying objects, including birds, balloons, and kites, using an illuminator transmitting orthogonal frequency-division multiplexing (OFDM) communication signals. A system consisting of a series of circular antennas, a signal processing module and a decision mechanism was proposed for the detection system, and a new fixed false alarm rate algorithm was implemented. In contrast, in [7], only one antenna was used to detect drones. The reference signal was generated by the remodulation of the surveillance channel. Synchronization, channel equalization, forward error correction and remodulation procedures were performed to generate the reference signal. An extensive cancellation algorithm (ECA) was recommended for eliminating multipath interference.

In [8], it was shown that microdrones can be detected using a UMTS based passive radar system. A two-step procedure for detection was proposed. The cross-ambiguity function of the signals was calculated using fast Fourier transform (FFT) and, prior to this calculation, by removing both the direct path and clutter in the surveillance signal using iterative algorithms. Although the conventional matched filter is statistically optimal under the assumption that noise is Gaussian, in the presence of impulsive effects, the suboptimality of the matched filter in the expansion of the main lobe and the suppression of the sidelobes may cause problems, and this would make the detection of low observable targets by applying the cross ambiguity function particularly challenging [1,2].

In addition to the conventional matched filtering method for target detection, other methods have also been proposed recently. For instance, compressed sensing (CS) has been used for target detection. The OFDM waveform was selected as the illuminator of opportunity in [9]. To produce a high resolution range-Doppler map for target detection, the MUSIC algorithm was first applied and then target detection was carried on using the CS concept. The CS algorithm was applied for target detection under the assumption of additive white Gaussian noise in [10], and it was noticed that the target detection performance was not optimal for low signal-to-noise ratio (SNR) inputs, because the reconstruction algorithms were not effective at low SNR. In [11], both the atomic norm and the $l_{1}$ norm were used within the CS framework to eliminate the direct signal, interference and clutter in passive radar systems, and to obtain super-resolution delay-Doppler estimation.

In [12], the sparse discrete fractional Fourier transform (SDFrFT) method, which has a much less computational complexity than the FFT-based cross ambiguity function method, was proposed. The SDFrFT algorithm improves target detection performance under Gaussian noise, producing maximum peaks even for low observable targets. The superiority of the SDFrFT algorithm against other algorithms in the detection of high-acceleration targets was proven by simulation and experimental scenarios.

Another alternative target detection approach is the track-before-detect (TBD) algorithm, which uses a range-Doppler map sequence without thresholding for synchronized detection and tracking. In TBD, the thresholding process in which observations are generated is eliminated, and the possibility of detecting a low SNR target is increased by preserving the weak signal information in the data [13]. In [14], a recursive Bayesian filter was applied to raw data obtained from an OFDM based passive radar, and the probability of target existence was calculated recursively in each delay-Doppler cell. In [15], the downlink of the illuminator was simulated as a code-division multiple access (CDMA) modulation signal and the dynamic programming (DP) algorithm was used for the detection of a weak target echo. To overcome the high computational load of the DP TBD method, a greedy algorithm with similar performance to that DP TBD, but with lower computational load is proposed in [16].

In the above works on CS, SDFrFT and TBD techniques, the background noise was modeled as Gaussian. However, in real world radar applications noise is often modeled non-Gaussian with distributions such as Weilbull, log-normal, Gaussian mixture model, K-distribution and α stable distribution [17]. The most important feature of the non-Gaussian models, which are encountered in real-world applications, is impulsiveness [18]. Therefore, this paper focuses on the impulsive noise model, which is among the non-Gaussian noise models. The probability density function (pdf) of an impulsive noise has a heavier tail than the pdf of a Gaussian noise, and with an impulsive noise, the detection performance of the CS and TBD methods in [11,19] degrades.

In [20], atmospheric radar clutter, as well as human-made interferences (e.g., power lines), is shown that produce impulsive noise in mobile communications. Note that demodulation errors also generate impulsive noise, and strong sidelobe interferences due to this added impulsive noise cause targets with low SNR to go undetected [11]. In the literature, multiple approaches have been proposed to model impulsive noise, such as α stable distribution [21], Gaussian mixture model [22] and generalized Gaussian model [23], with approach to delay and Doppler estimation. To overcome the problem caused by impulsive noise, fractional lower order statistics-based algorithms were used in [24,25,26,27,28]. However, the performance of these algorithms degrades when the impulsive nature of the noise increases [29].

The detection of low radar cross-section (RCS) targets in the presence of impulsive noise is a very challenging problem, especially for passive radars with low power and resolution due to their waveforms and illuminator properties [30]. The aim of this paper is to create a framework that solves this challenging problem by minimizing the weighted $l_{1}$ and $l_{2}$ norms. Using the $l_{1}$ and the $l_{2}$ norms as a weighted form aims to provide a general solution framework while preserving the superiority of these two norms with different environmental conditions. We consider the case where the noise is modeled as an α stable distribution, which is a commonly used model for impulsive noise [18,21,25]. In this paper, a two-stage algorithm is proposed. In the first stage, the α parameter, which determines the degree of the impulsivity of the noise is estimated. In the second stage, a minimization function is formulated for weighting the $l_{1}$ and $l_{2}$ norms based on the estimated α parameter. Range and Doppler parameters of the targets can be estimated by solving this minimization problem.

In this paper, the aim is to fill certain gaps in previous passive radar target detection studies. The specific contributions at this paper as follows:

- Although a large number of studies focus on passive radars with mobile communication signals such as UMTS, these studies assume that the communication signal is demodulated perfectly. While this a valid assumption, it overlooks the errors caused by less than perfect demodulation. The demodulation causes an impulsive noise on the received signals, which will deteriorate the detection performance. Unlike other studies, perfect demodulation assumption is dropped in this study to provide an approach that is better suited for real life applications.
- The major novelty of the proposed algorithm is to construct a new objective function that solves the problem of detecting low RCS targets in the presence of impulsive noise by weighting the $l_{1}$ and $l_{2}$ norm. We aim to provide a general solution framework for the detection problem using the $l_{1}$ and the $l_{2}$ norms as a weighted form while preserving the superiority of these two norms in different environmental conditions. Thus, a new filtering concept is created by adopting weighted $l_{1}$ and $l_{2}$ norm optimization.
- There are studies in the literature where delay-Doppler is estimated by filters defined in the $l_{p}$ space. In these studies, the cross ambiguity function is calculated for a fixed p-value. Whereas, the new filtering concept that can adaptively adjust the filter characteristics according to the impulsiveness level of the noise is proposed in this paper. Adaptive adjustment of the proposed filtering concept will prevent performance degradation of the proposed algorithm in environments with different noise characteristics.
- In the few existing studies where the noise is modeled as impulsive, the performance analysis is limited to simulation results. However, in this paper, the performance analysis of the proposed algorithm under impulsive noise is also carried out on real data from an operation at passive radar platform.

This paper is organized as follows. We define signal and noise models of the passive radar system in Section 2. Conventional methods used for performance comparison are briefly reviewed in Section 3. In Section 4 the proposed method is presented along with the background of the method on $l_{p}$ norm and estimation of the α parameter. In Section 5 the detection results using both simulated and experimental data are given. Finally, we summarize the conclusions in Section 6.

## 2. Signal and Noise Models

### 2.1. Signal Model

**Figure 1:**
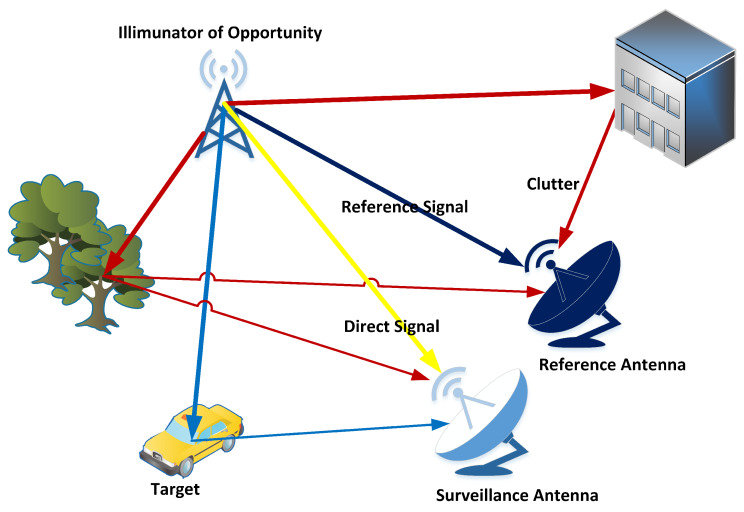
A common scenario for passive radar systems.

This paper uses a two-channel model, consisting of reference and surveillance channels, which is traditionally used in passive radar systems as shown in Figure 1 [1].

The data collection model is composed of two antennas, one of which is positioned directly to receive the reference signal from the base station while the other collects the reflected signal from the surveillance area. The signal reaching the reference channel is defined as

(1) $$s_{ref}\left(t\right)=\beta_{0}x\left(t-\tau_{0}\right)+w_{ref}\left(t\right)$$

where x(t) is the signal transmitted from the illuminator and $\beta_{0}$ is the constant complex attenuation coefficient for the $\tau_{0}$ round trip time and $w_{ref}\left(t\right)$ is additive noise in the reference channel. The signal that reaches the surveillance antenna consists of four parts. The first one is the direct path signal ($s_{dps}\left(t\right)$), the second is composed of the echo signals reflected from moving targets ($s_{trgt}\left(t\right)$), the third is composed of clutter signals received from stationary objects in the background ($s_{clutter}\left(t\right)$) and the last one is an additive noise signal ($w_{surv}\left(t\right)$). Then, the general expression for the received signal can be written as

(2) $$s_{surv}\left(t\right)=s_{dps}\left(t\right)+s_{trgt}\left(t\right)+s_{clutter}\left(t\right)+w_{surv}\left(t\right)$$

or, equivalently,

(3) $$s_{surv}\left(t\right)=\gamma_{0}x\left(t-\tau_{0}\right)+\sum_{k=1}^{K}\beta_{k}x\left(t-\tau_{k}\right)e^{j2\pi f_{dk}t}+\sum_{m=1}^{M}\gamma_{m}x\left(t-\tau_{m}\right)e^{j2\pi 0t}+w_{surv}\left(t\right)$$

where K is the number of target sources, M is the number of stationary clutter sources, $\beta_{k}$ is the complex attenuation coefficient for the received signal with $\tau_{k}$ delay, $\gamma_{m}$ is the stationary clutter complex gain factor and $\gamma_{0}$ is the gain of the direct signal collected with the sidelobes of the antenna. Further, $f_{dk}$ is the Doppler shift of the *k*th target and $w_{surv}\left(t\right)$ is the additive noise in the surveillance channel. To write the signals in discrete-time, the following transformations in Equation (4) is applied:

(4) $$\begin{array}{cc} t & =nT_{s}\\ f_{d} & =\frac{kf_{s}}{N}=v_{k}\\ f_{s} & =\frac{1}{T_{s}} \end{array}$$

where $T_{s}$ is the sampling period and N is the number of samples. Then, the discrete-time signals are expressed as

(5) $$s_{ref}\left(n\right)=\beta_{0}x\left(n-D_{0}\right)+w_{ref}\left(n\right)$$

(6) $$s_{surv}\left(n\right)=\sum_{k=1}^{K}\beta_{k}x\left(n-D_{k}\right)e^{j2\pi v_{k}t}+\sum_{m=0}^{M}\gamma_{m}x\left(n-D_{m}\right)+w_{surv}\left(n\right)$$

where *n* represents the discrete-time index, $D_{m}$ is the sample delay and $v_{k}$ is the normalized Doppler shift of the corresponding path.

### 2.2. Noise Model

The Gaussian noise model is commonly used in signal processing and communication applications for various reasons. First, even if the distribution of a data set is not normal, the distribution of the mean of the samples from this distribution approximated by the normal distribution due to the Central Limit Theorem. Second, this distribution can be approximated using the first two order moments, and an analytical solution can be obtained based on approximation Gaussian noise model.

The Gaussian noise model is generally used in passive radars as well [3,4,5,6,7,8,9,10,11,13,14,15,16]. However, these methods are not optimal in real world problems with non-Gaussian noise. This is due to the fact that the noise is non-Gaussian in real world problems, but, even so, it is solved under the assumption that it is Gaussian. One of the non-Gaussian noise model classes is the impulsive noise model and, in this paper, the α stable noise model [18,21,25,31] is chosen as the noise model.

#### α Stable Noise Model

The α stable noise model shows more impulsive characteristics compared to the Gaussian noise model, due to the heavier tail in its probability density function. This allows for the generation of more noise outlier samples than what is possible with the Gaussian noise model. The α stable is commonly used for impulsive noise because of the stability of the α stable distribution (i.e., the linear combination of two different random variables also has the same distribution [18]). While modeling the ’spikes’, the noise that occur over time can be modeled with this distribution [21].

Although we cannot define a closed form expression for the pdf of α stable distribution, we can define its characteristic function as

(7) $$\varphi \left(t\right)=e^{\left(j\mu t-\gamma {\left|t\right|}^{\alpha }\left[1+j\beta \mathrm{sign}\left(t\right)w\left(t,\alpha \right)\right]\right)}$$

where

(8) $$w\left(t,\alpha \right)=\left\{\begin{array}{ccc} \tan\frac{\alpha \pi }{2} & \;\mathrm{for} & \alpha \neq 1\\ \frac{2}{\pi }\log\left(t\right) & \;\mathrm{for} & \alpha =1 \end{array}\right.$$

and

(9) $$\mathrm{sign}\left(t\right)=\left\{\begin{array}{ccc} -1 & \;\mathrm{for} & t<0\\ 0 & \;\mathrm{for} & t=0\\ 1 & \;\mathrm{for} & t>0 \end{array}\right.$$

The α is the characteristic exponent parameter with 0<α≤2 that determines the heaviness of the tails of the distribution. When the α value is close to zero, the impulsiveness of the noise increases. In the above, location parameter μ span is (−∞<μ≤∞), μ is equal to the mean noise value when 1<α≤2 and the median when 0<α≤1. The dispersion parameter γ can be greater than zero, and it measures the spread of the distribution. The last parameter, β−1≤β≤1, is the symmetry parameter and the α distribution is called symmetric α-stable (SαS) for β=0 [31].

**Figure 2:**
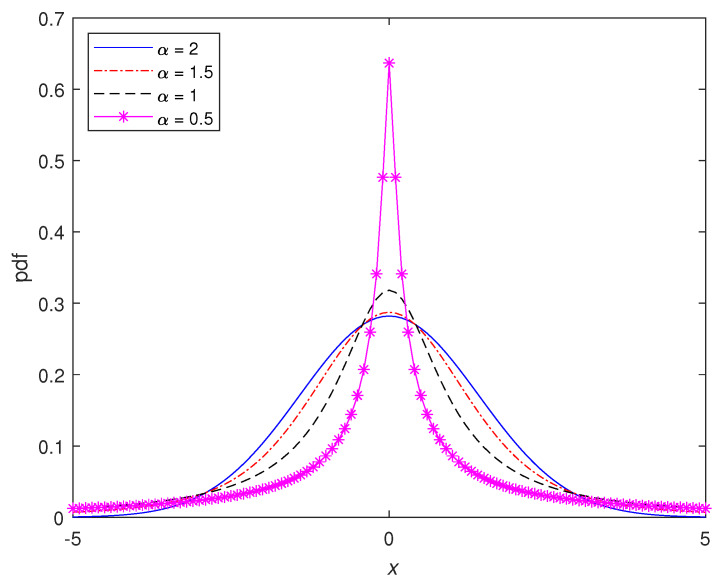
Pdf of symmetric α-stable distributions for the different α values.

Figure 2 shows the pdf of the symmetric α stable distribution for different values at α with zero location. The α characteristic exponent is selected as 0.5, 1 and 2 with location parameter μ=0, dispersion parameter γ=1 and symmetry parameter β=0. It can be seen from Figure 2 that when the value of α is equal to 2, the distribution corresponds to a normal distribution, and as the α value gets smaller, the tail becomes heavier.

**Figure 3:**
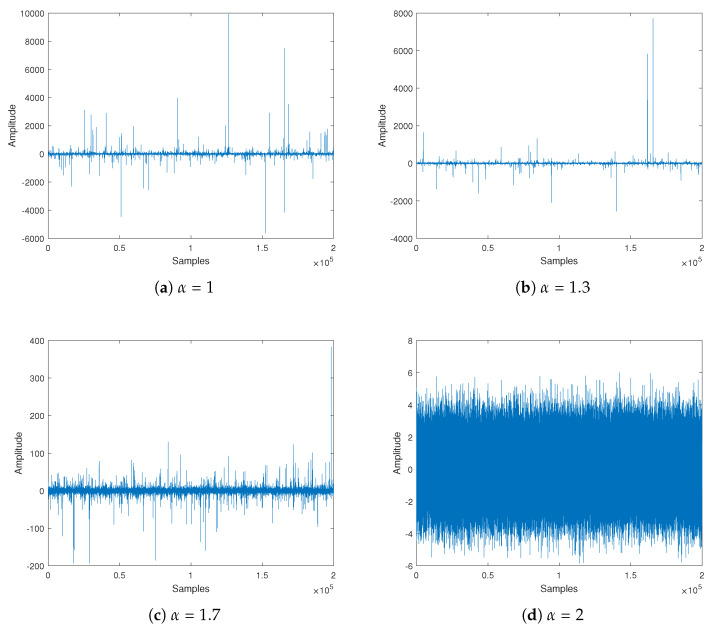
Sample time series of random variables with a symmetric α stable distribution for different α values.

Figure 3 presents the sample time series of random variables, which have the symmetric α stable distribution for different at α values with zero location. The α characteristic exponent is selected as 1, 1.3, 1.7 and 2 with location parameter μ=0, dispersion parameter γ=1 and symmetry parameter β=0. From Figure 3, we can see how the impulsive nature of the noise changes with α. The number and amplitude of the spiky peaks in the sample time series increase as the value of the α parameter decreases.

## 3. Conventional Methods

### 3.1. Cross Ambiguity Function

Cross-ambiguity function (CAF), traditionally used to generate range-Doppler maps represents the output of the matched filter. The calculation of this function is the basis for estimating the time delay and frequency shift between two waveforms with additional noise [32]. The cross-ambiguity function, a function of time delay and Doppler shift, is calculated during the integration time, and uses complex signals from the reference and the surveillance antennas. The calculation of the cross-ambiguity function in continuous time is given by

(10) $$CAF\left(\tau ,f_{d}\right)=\int_{0}^{T_{int}}s_{ref}\left(t\right)s_{surv}^{*}\left(t+\tau \right)e^{-j2\pi f_{d}t}dt$$

For the estimation of time delay and Doppler shift, which are the common components of $s_{ref}$ and $s_{surv}$ complex signals, the τ and $f_{d}$ parameters are sought to form the peaks of the cross-ambiguity function. The surveillance signal is shifted in two steps when calculating the cross-ambiguity function. In the first step, the surveillance signal is shifted horizontally in the time domain for the specified frequency, and at these shifted points the surveillance signal is multiplied by the reference signal and the result is summed. In the second step, the surveillance signal is shifted vertically in the frequency domain for a certain time point, and the multiplication and addition operations are repeated as in the first step.

For the horizontal shifting case, in which the Doppler shift is zero, the evaluation of the cross-ambiguity function becomes the calculation of the standard cross-correlation function. For non-zero values of the Doppler shift, the vertical shifting can be seen as a correlation calculation after replacing the entire spectrum in the reference signal with the Doppler shift values. The peak values are reached when the shift values in both the time and the frequency domain fully compensate for the time and Doppler shift from the surveillance signal.

For time delay and Doppler shift estimation, the τ and $f_{d}$ parameters will be searched to find the peak of the discretized signal using the function in Equation (10). Basic sampling steps must be applied for the discretization process in both time and frequency domains. In the time domain, a discrete time signal can be obtained by sampling a continuous time signal uniformly at sampling period $T_{s}$. In the frequency domain, the frequency samples are acquired by using transformation of $f_{d}=kf_{s}/N$ where *k* is an integer and $f_{s}=1/T_{s}$. The discrete form of the cross-ambiguity function is written as

(11) $$\begin{array}{cc} CAF\left(m,k\right) & =\sum_{n=0}^{N-1}s_{ref}\left(n\right)s_{surv}^{*}\left(n+m\right)e^{-j2\pi \frac{kf_{s}}{N}nT_{s}}\\ \; & =\sum_{n=0}^{N-1}s_{ref}\left(n\right)s_{surv}^{*}\left(n+m\right)e^{-j2\pi \frac{kn}{N}} \end{array}$$

### 3.2. Fractional Lower Order Statistics Cross Ambiguity Fuction

Since the CAF cannot cope with outliers caused by impulsive noise, performance losses in the presence of impulsive noise are mitigated using a method based on fractional lower order statistics (FLOS) in [24]. In a manner similar to that of the CAF, this method for joint delay and Doppler estimation is briefly formulated as follows:

(12) $$FLOSCAF\left(m,k\right)=\langle {\left(s_{ref}\left(n\right)\right)}^{\left(x\right)},e^{-j2\pi k\frac{kf_{s}}{N}t}{\left(s_{surv}\left(n+m\right)\right)}^{\left(y\right)}\rangle$$

Using the definition of the inner product, $FLOSCAF\left(m,k\right)$ can be written as

(13) $$FLOSCAF\left(m,k\right)=\frac{1}{L_{2}-L_{1}}\sum_{n=L_{1}+1}^{L_{2}}\left({\left(s_{ref}\left(n\right)\right)}^{\left(x\right)}{\left(s_{surv}^{*}\left(n+m\right)\right)}^{\left(y\right)}e^{-j2\pi k\frac{kf_{s}}{N}t}\right)$$

where $L_{1}=\max\left(0,-k\right)$, $L_{2}=\min\left(N-k,N\right)$. Futhermore, *x* and *y* should be selected between zero and half of α. The estimated delay ($\hat{D}$) and Doppler ($\hat{\upsilon }$) values are found by searching the peaks of the absolute value of the $FLOSCAF\left(m,k\right)$ as

(14) $$\left\{\hat{D},\hat{\upsilon }\right\}=\underset{m,k}{\arg\max}\left|FLOSCAF\left(m,k\right)\right|$$

The FLOSCAF method was used to eliminate the target detection performance decrease in the presence of impulse noise of the CAF method. However, this method has difficulty in dealing with the problem resulting from the fact that the impulsive noise distribution produces more outliers when the probability density function has a heavier tail [29]. To eliminate this problem, a new optimization based range-Doppler map generation algorithm is proposed in this paper.

## 4. Proposed Algorithm

In this section, first, we provide the motivation for using the $l_{1}$ and the $l_{2}$ norms in the proposed method. The minimization problem in the proposed method is defined by weighting the $l_{1}$ and $l_{2}$ norms. The reason of selecting these two norms is explained through the solution approach of these norms for a simple optimization problem. The $l_{p}$ norm approach to the minimization problem is given by

(15) $$\begin{array}{cc} \min_{x}{∥y-\mathrm{Ax}∥}_{p}^{p}\; & =\min_{x}\left(obj_{p}\left(r\right)\right)\\ \; & =\min_{x}\left({\left|r_{1}\right|}^{p}+{\left|r_{2}\right|}^{p}+\ldots +{\left|r_{N}\right|}^{p}\right)\\ \mathrm{subject}\;\mathrm{to} & \;r=y-\mathrm{Ax} \end{array}$$

where $A\in R^{NxM}$ with rank (A)=N, $y\in R^{N}$ vector and r is residual. The $l_{p}$ norm approach given for the solution of the above problem is a generalization of the approach we use in our proposed algorithm. Therefore, the $l_{p}$ norm properties shown through the solution approach of this simple problem help define the objective function in Equation (17).

**Figure 4:**
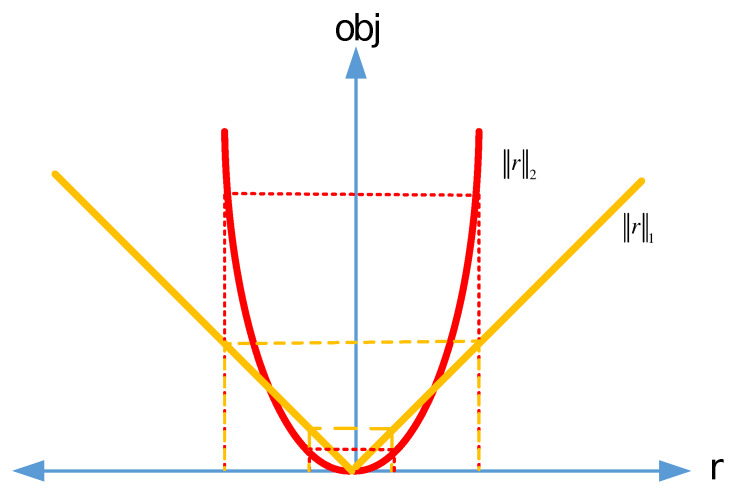
Responses of $l_{1}$ and $l_{2}$ norm approaches vs. residual.

The $l_{p}$ norm minimization with 0≤p<1 is NP hard, and optimal solutions are not feasible [33]. Moreover, although the $l_{p}$ norm with 0≤p<1 produces better results in sparse problems, the non-convex optimization problem in this interval makes the problem more difficult to solve [34]. Therefore, we will focus on the p=1 and p=2 norms, which have lower computational complexity than 0≤p<1. As seen from Figure 4, the responses of $l_{1}$ and $l_{2}$ norm approach the r value defined as $obj_{1}\left(r\right)=\left|r\right|$ and $obj_{2}\left(r\right)={\left|r\right|}^{2}$, respectively. For example, when r is equal to 1, both approaches produces same response. However, it is seen in Figure 4 that $obj_{1}\left(r\right)\ll obj_{2}\left(r\right)$ for large values of r (e.g.,r≫1), whereas $obj_{1}\left(r\right)\gg obj_{2}\left(r\right)$ for small values of r (e.g.,r≪1).

**Figure 5:**
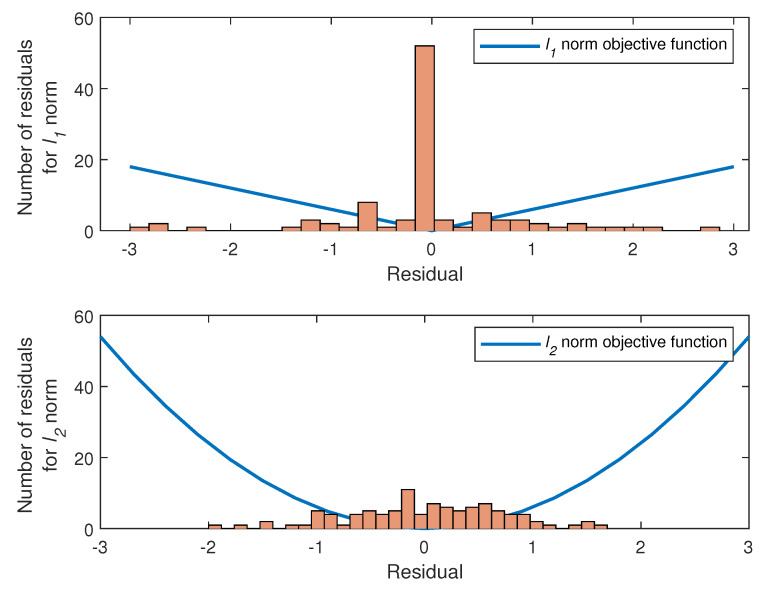
Histogram of residual amplitudes for $l_{1}$ and $l_{2}$ norm solutions

We can give an example to demonstrate how this feature works. For the matrix $A{\in R}^{100x50}$ and $y{\in R}^{100}$ vector which are chosen at random, the amplitude histogram for r obtained by calculating the approximate solutions of Ax≈y using $l_{1}$ and $l_{2}$ norm approaches is presented in Figure 5.

Figure 5 shows that the residuals obtained in the optimal solution of $l_{1}$ norm are zero or close to zero, since the $l_{1}$ norm approach assigns a higher amplitude response to the smaller residuals than the $l_{2}$ norm approach. On the contrary, $l_{2}$ norm approach produces uniform residuals as a result of the optimal solution of the $l_{2}$ norm, because the $l_{2}$ norm approach puts more weight on the residuals than the $l_{1}$ norm approach [35]. From the results we deduce that the use of $l_{1}$ and $l_{2}$ norms in the objective function in Equation (17) will result in different solutions for different noise distributions. If the noise has a Gaussian distribution, $l_{2}$ norm yields the optimal solution [2]. This means that the $l_{2}$ norm approach is effective when there exist a very small number of outliers. However as the impulsive nature of the noise increases with many outliers as shown in Figure 5, the $l_{1}$ norm approach yields better solution [35]. This helps conclude that the $l_{1}$ norm approach is more robust than the $l_{2}$ norm approach in dealing with outliers.

Note that the $obj_{1}\left(r\right)$ and $obj_{2}\left(r\right)$ objective functions defined using the $l_{1}$ and the $l_{2}$ norms are convex. The weighted sum of these objective functions preserves

(16) $$obj=w_{1}{obj}_{1}+w_{2}{obj}_{2}$$

convexity as long as the weights $w_{i}\geq 0$. This feature guarantees that the optimization problem defined in Equation (17) is a convex optimization problem. The fact that the problem is a convex optimization problem provides some advantages as follows: First, the local optimum is actually global optimum. Second, preserving the convexity of the optimization problem enables that the function is optimized reliably and efficiently with the algorithms to be applied [35,36].

A new algorithm is proposed in this paper for the generation of range-Doppler map for delay and Doppler estimation in the presence of impulsive noise by taking advantage of the $l_{1}$ and the $l_{2}$ norm approaches. The proposed algorithm transforms the calculation of the cross ambiguity function from the Hilbert space to the $l_{p}$ space, and this idea was inspired by [37]. In contrast with [37], in this paper, a new filter concept that adaptively adjusts itself by determining the impulsivity level of impulsive noise in the environment is proposed. Furthermore, an optimization problem is define to guarantee convexity by using a combination of $l_{1}$ and $l_{2}$ norms that are known to have different advantages in different environments. In the new algorithm, the calculation of cross ambiguity function in $l_{p}$ space is preferred because this increases time and Doppler resolution and provides better sidelobe suppression compared to the traditional calculation in the Hilbert space [37].

A two-step optimization strategy is used to estimate the range and Doppler parameters. A global minimizer β value is sought for the given time delays *m* and the $V_{k}$ Doppler shift in the first step. The optimization function to find this value is defined as

(17) $$h\left(m,k\right)=\min_{\beta }\left(w_{1}{∥s_{\mathrm{surv}}-\beta \theta \left(V_{k}\right)s_{\mathrm{ref}}\left(m\right)∥}_{1}+w_{2}{∥s_{\mathrm{surv}}-\beta \theta \left(V_{k}\right)s_{\mathrm{ref}}\left(m\right)∥}_{2}\right)$$

where $\theta \left(V_{k}\right)$ is a diagonal matrix given as

(18) $$\theta \left(V_{k}\right)=\left[\begin{array}{cccc} e^{j2\pi V_{k}0} & 0 & \cdots & 0\\ 0 & e^{j2\pi V_{k}1} & 0 & 0\\ \vdots & 0 & \ddots & 0\\ 0 & 0 & 0 & e^{j2\pi V_{k}\left(N-1\right)} \end{array}\right]$$

and $w_{1}$ and $w_{2}$ are the weighting factors used for the $l_{1}$ norm and the $l_{2}$ norm respectively. There is no closed form solution for $l_{p}$ norm approximations except for the $l_{2}$ norm approximation. For this reason, a closed form solution cannot be given for the optimization problem defined in Equation (17).

The values of the weighting factors quantize inputs of $l_{1}$ and $l_{2}$ norms in the minimization problem given in Equation (17). Determination of the weighting factors has a major influence on the algorithm. The α parameter of α stable distribution, which is the noise model used in the signal definition in Section 2.1, is needed in the determination of the weighting factors ($w_{1}$,$w_{2}$). This is because the parameter that determines the impulsiveness of the noise is α parameter as can be seen in Figure 2. For α values varying 1 and 2, the impulsive characteristic of the noise increases as α approaches 1; contrarily, the impulsiveness decreases as α gets closer to 2. The key to determining the weights in the proposed algorithm is the estimation of the α parameter, which determines the impulsiveness level of the noise.

We can give some details on the method for estimating this parameter. Parameters estimation techniques [38,39,40] with α stable distribution have a high computational load and requiring lookup table and show low level convergence [41]. The quantiles method is used in estimation of the parameters of α stable distribution [42,43]. For the estimation of the stability, skewness, scale and location parameters of α stable distribution, quantiles (${\hat{q}}_{0.05},{\hat{q}}_{0.25},{\hat{q}}_{0.5},{\hat{q}}_{0.75},{\hat{q}}_{0.95}$) are determined and statistics of these quantiles (${\hat{\vartheta }}_{\alpha },{\hat{\vartheta }}_{\beta }$) are computed as

(19) $$\begin{array}{cc} {\hat{\vartheta }}_{\alpha } & =\frac{{\hat{q}}_{0.95}-{\hat{q}}_{0.05}}{{\hat{q}}_{0.75}-{\hat{q}}_{0.25}}\\ {\hat{\vartheta }}_{\beta } & =\frac{{\hat{q}}_{0.95}+{\hat{q}}_{0.05}-2{\hat{q}}_{0.5}}{{\hat{q}}_{0.95}-{\hat{q}}_{0.05}} \end{array}$$

where $\hat{q}$ is the given data and the estimation of α and β parameters is a function of these statistics given by

(20) $$\begin{array}{cc} \hat{\alpha }=\psi_{1}\left({\hat{\vartheta }}_{\alpha },{\hat{\vartheta }}_{\beta }\right) & \hat{\beta }=\psi_{2}\left({\hat{\vartheta }}_{\alpha },{\hat{\vartheta }}_{\beta }\right) \end{array}$$

The $\hat{\alpha }$ and $\hat{\beta }$ parameters are estimated by interpolating the lookup tables defined in [43]. The estimation of the skewness parameter is calculated after the estimation of α and β parameters. This is because ${\hat{q}}_{0.25},{\hat{q}}_{0.75}$ quantiles as well as a function of α and β parameter estimations are used in the estimation of the skewness parameter as

(21) $$\hat{\gamma }=\frac{{\hat{q}}_{0.75}-{\hat{q}}_{0.25}}{\psi_{3}\left(\hat{\alpha },\hat{\beta }\right)}$$

where $\psi_{3}\left(\hat{\alpha },\hat{\beta }\right)$ function is obtained using linear interpolation by lookup table [43]. An intermediate stage is required to estimate the location parameter μ. In this intermediate step, the ξ parameter used in estimating the location parameter μ is calculated as

(22) $$\xi =\left\{\begin{array}{cc} \mu +\beta \gamma \tan\frac{\pi \alpha }{2}, & \;\alpha \neq 1\\ \mu , & \;\alpha =1 \end{array}\right.$$

The ξ parameter is estimated using ${\hat{q}}_{0.5}$ and $\psi_{5}\left(\hat{\alpha },\hat{\beta }\right)$ function, which is calculated by interpolating on [43] as

(23) $$\hat{\xi }={\hat{q}}_{0.5}+\hat{\gamma }\psi_{5}\left(\hat{\alpha },\hat{\beta }\right)$$

Finally, the location parameter μ is estimated with the ξ parameter defined in Equation (23) as

(24) $$\hat{\mu }=\hat{\xi }-\hat{\beta }\hat{\gamma }\tan\frac{\pi \hat{\alpha }}{2}$$

To avoid using of the lookup tables in the quantiles method, empirical characteristic function (ECF) has been proposed [44,45]. Although it is not necessary to initialize the ECF method with the parameters estimated by the quantiles method presented by Equations (19)–(24), it is recommended for more accurate estimates. In this paper, the initial parameter estimates are obtained using the quantiles method for the given sample data and then the sample data are standardized using the initial value of the skewness parameter β(0). To correct the α and γ parameter estimates at the beginning of the iteration, the least squares regression is applied against the ECF, and then the data are rescaled using the estimated scale value. In the next step, the regression process applied to correct the α and γ estimates in the previous step is also performed to correct of the β and μ parameters. The steps are continued until the convergence. Assuming that the sample data $\left\{x_{1},x_{2},\ldots ,x_{N}\right\}$ are independent and identically distributed, the estimation of the characteristic function can be written based on the law of large numbers as [44,45]

(25) $$\hat{\varphi }\left(t\right)=\frac{1}{N}\sum_{i=1}^{N}e^{jtx_{i}}$$

where *N* is the number of sample. Some observations should be followed in the regression method proposed in [44]. The first step is to obtain the logarithm of the characteristic functio in Equation (7) using

(26) $$\log\left(-\log{\left|\varphi \left(t\right)\right|}^{2}\right)=\log\left(2\gamma^{\alpha }\right)+\alpha \log\left(t\right)$$

The real and imaginary parts of characteristic function in Equation (7) for α≠1 can be respectively shown to be [44]

(27) Reφt=e−γtαcosμt+γtαβsignttanπα2Imφt=e−γtαsinμt+γtαβsignttanπα2

Using the above real and imaginary parts of the characteristic function, the α and γ parameters can be estimated by applying the regression method after calculating the empirical characteristic function for *t*. That is,

(28) arctanImφtReφt=μt+γtαβsignttanπα2

and the α and γ parameters are estimated by regressing $y=\log\left(-\log{\left|\varphi \left(t\right)\right|}^{2}\right)$ on $w=\log\left(t\right)$ in the model

(29) $$\begin{array}{cc} y_{k}=m+\alpha w_{k}+\varepsilon_{k} & k=1,2,\ldots ,K \end{array}$$

where $m=\log\left(2\gamma^{\alpha }\right)$, $w_{k}=\log\left(t_{k}\right)$ and $\varepsilon_{k}$ is an error term. The real data set tk=πk25 for *k* = 1,2,…,*K* can be computed in [44].

After estimating the α parameters, estimated parameter value is converted into a weighting factor ranging from 0 to 1. The weighting functions proposed in this paper are given by

(30) $$\begin{array}{c} w_{2}=\frac{e^{\hat{\alpha }}-e^{1}}{e^{2}-e^{1}}\\ w_{1}=1-w_{2} \end{array}$$

Then, in the second step, based on all possible *m* and *k* values, $h\left(m,k\right)$, as defined in (17) is obtained by maximizing

(31) $$WCAF\left(m,k\right)=\left({∥s_{\mathrm{surv}}∥}_{1}-h\left(m,k\right)\right)$$

**Figure 6:**
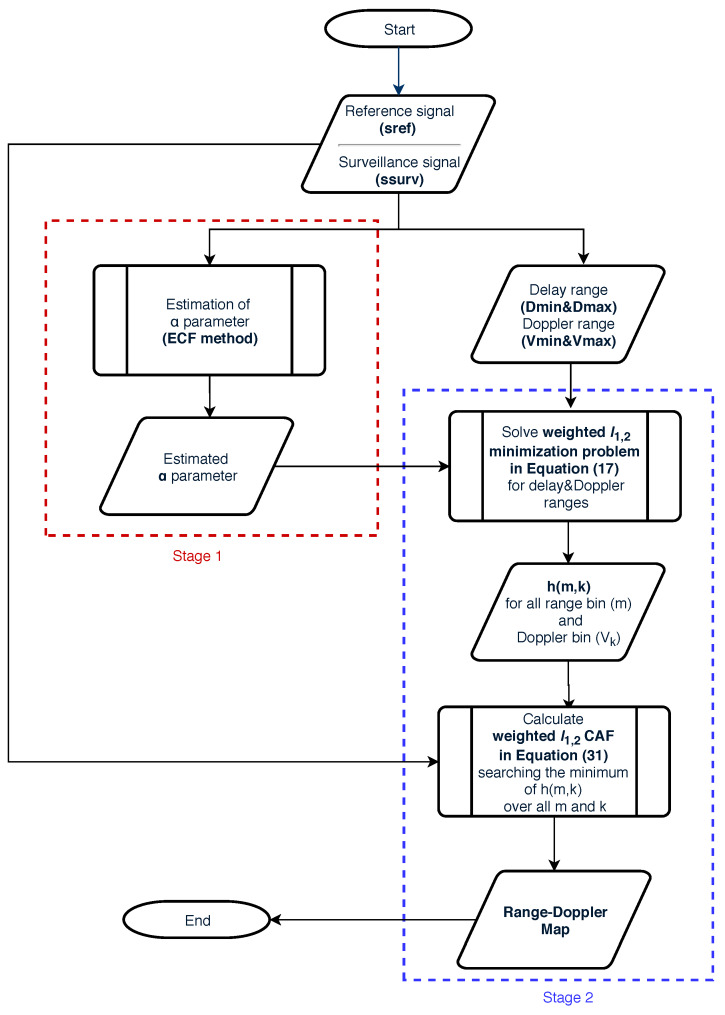
Flowchart of the proposed algorithm.

We can summarize our range-Doppler map generation algorithm using the flowchart in Figure 6.

To solve the minimization problem in Equation (17) we used the primal-dual symmetric interior point algorithms [46]. One of the main issues to consider when choosing the algorithm is the computational complexity [36]. Computational complexity is important in an optimization problem in terms of showing how fast the completion time of our proposed algorithm increasing especially with as dimension of problem samples. In our convex optimization problem, the computational complexity grows moderately with the dimension of the problem and the desired level of accuracy, while the complexity of the non-convex problems increases rapidly with the dimension of the problem and the desired level of accuracy [47]. Many methods are proposed in the literature for the solution of convex optimization [35,36,47]. Our optimization problem defined in Equation (17) can be calculated with the gradient descent method [35] and the center of gravity method [36], and if the problem is solved by using these methods, the computational complexity becomes O(Nlog1/ϵ) where ϵ∈(0,1) represents the desired accuracy [36]. However, if we solve the problem with the primal-dual symmetric interior point method, the computational complexity decreases to $O(\sqrt{N}\log N/\epsilon )$ [48]. We implemented that method using CVX package for specifying and solving convex programs in Equation (17) [49].

## 5. Simulation and Experimental Results

### 5.1. Simulation Results

First, a passive radar simulator is used to compare the proposed algorithm with the CAF and the FLOSCAF methods. The reference and surveillance signals are generated in the passive radar simulator using the signal models in Equation (5) and (6). The target attenuation levels used during the generation of the surveillance signal depend on the RCS values of the targets. The noise components, which are additive parts of the signals, are produced according to an α stable distribution in these scenarios. These noise components have a symmetric α stable distribution with zero location.

#### 5.1.1. Performance Criteria

**Figure 7:**
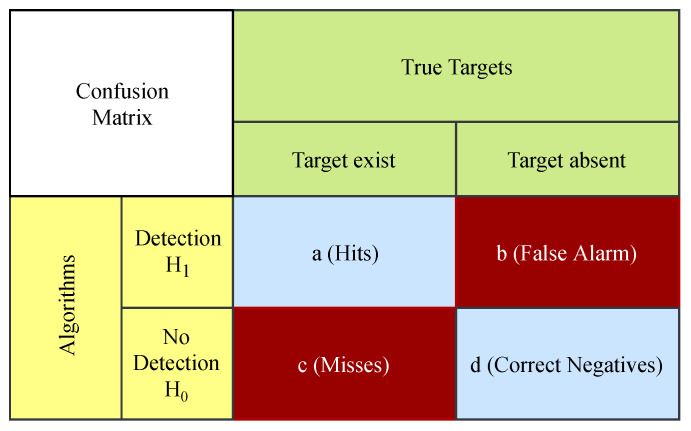
The structure of the confusion matrix.

Two performance metrics are used to compare the performance of algorithms in different scenarios. Our first performance metric is the probability of detection (PoD). First, confusion matrices are built to find the PoD scores of the algorithms. With the creation of the confusion matrix, metrics such as PoD, false alarm rate, threat score can be obtained to compare the detection performance of the algorithms. The structure of the confusion matrix used in this study is presented in Figure 7.

Two maps are compared to define the confusion matrix. The first map is the detection map produced by the algorithms, and the other is the true target map where targets are in the correct range and Doppler positions. Each test cell on the detection map is compared to the cell at the same point in the true targets map. If the target exists in the cell on the true target map and that target is detected in the same cell on the detection map, the value of ***a*** is increased by 1, in the contrary case the value of ***c*** is increased by 1. Similarly, if there is no target in the cell on the true target map and that target is detected at the same point on the detection map, the value of ***b*** is increased by 1. Otherwise, the value of ***d*** is increased by 1. After creating the confusion matrix, the PoD is calculated as

(32) $$\mathrm{PoD}=\frac{a}{a+c}$$

The second metric used as the performance criterion is the root mean square error (RMSE). In calculating the RMSE, only the cases where the target has been detected ($H_{1}$) are taken into account. RMSE is computed as

(33) $$RMSE=\sqrt{\frac{1}{N_{mc}}\sum_{i=1}^{N_{mc}}\frac{1}{\left(a+c\right)}\sum_{j=1}^{a+c}\left({\left(D_{j}^{\left(i\right)}-{\hat{D}}_{j}^{\left(i\right)}\right)}^{2}+{\left(V_{j}^{\left(i\right)}-{\hat{V}}_{j}^{\left(i\right)}\right)}^{2}\right)}$$

where $N_{mc}$ is the number of Monte Carlo runs. As seen from Equation (33), the Euclidean distance is calculated between the cell positions of true targets on the true targets map and the cell positions of the estimated targets on the detection map. Therefore, the calculated error unit is the cell distance.

#### 5.1.2. Scenarios

Table 1 presents the range, Doppler and RCS values of 10 different targets created in the passive radar simulator to analyze detection performance of the algorithms. The reference and surveillance signals are created by the passive radar simulator. The passive radar simulator generates the surveillance signal by using a time delay that corresponds to each target’s distance, a Doppler shift that corresponds to each target’s velocity, and each target’s RCS value. The matched filter output of these reference and echo signals, which are not exposed to any impulsive effects, gives the time delay and Doppler shift values of the true targets in Table 1.

**Figure 8:**
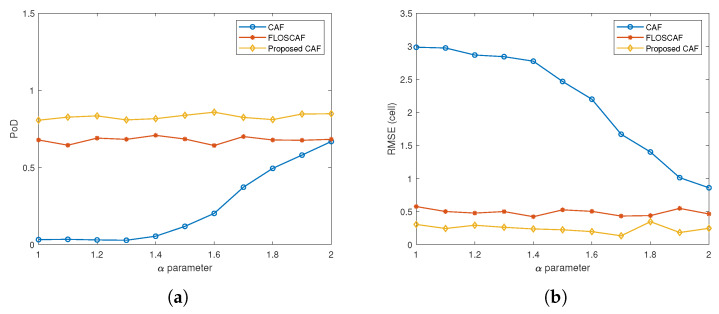
(**a**) Probability of Detection (PoD) comparison for different algorithms (RCS = 0.5 m^2^) (**b**) Root Mean Square Error (RMSE) comparison for different algorithms (RCS = 0.5 m^2^).

The simulation scenarios are carried out for 10 different targets with different Doppler and range values, the details of which are given in Table 1. The detection performance of the algorithms is compared in three different scenarios created for 10 different targets with RCS values of 0.5, 1 and 2 m^2^. Simulations are run 50 times for different α stable distributed impulsive noise components with different characteristic exponent value varies from 1 to 2 at intervals of 0.1 in each scenario. First, eight targets with different Doppler shifts and time delays given in Table 1 are created from the passive radar simulator. Then, new targets are added to the current situation in the scenario to make the problem more challenging. The added targets are set to be in the same range and neighbour Doppler positions as some existing ones to make the scenarios more complicated. The first added target is located in the same range cell as target 4 and Doppler cell 0. The second added target is located in the same range cell as target 7, and Doppler cell 5, which is one cell neighbour Doppler position relative to target 7. A total of 10 targets are eventually created by using passive radar simulator. Finally, the proposed weighted $l_{1,2}$ CAF, the CAF and the FLOSCAF algorithms are applied to the surveillance and reference signals generated by the simulator for obtaining the range-Doppler maps. In these scenarios, a two-step detection algorithm has been implemented. In the first step, a threshold value is determined by calculating the average power through the Doppler cells corresponding to each range value. Then the Doppler cells that are above the threshold value at the relevant range value are marked. In the second step, a threshold value is determined by calculating the average power along the range cells corresponding to each Doppler value. Afterward, the range cells above the threshold value of the Doppler value are marked. If the test cell is marked in both steps, the target is detected in that cell. The RCS value of all the targets is 0.5 m^2^ in the first scenario and Figure 8 shows the PoD and RMSE values for different α parameter values used to compare the detection performances of three different algorithms. The proposed weighted $l_{1,2}$ CAF as seen from Figure 8a has a higher PoD value compared to the other two methods in the whole α parameter range. The average PoD value over the entire α range is 0.237 for CAF, 0.678 for FLOSCAF, and 0.828 for the proposed algorithm. Likewise, when the RMSE values in Figure 8b are taken into consideration, it can be said that the proposed method has a lower RMSE value in the whole α range.

**Figure 9:**
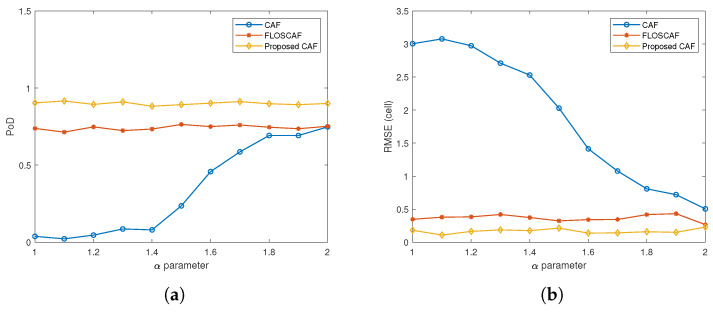
(**a**) PoD comparison for different algorithms (RCS = 1 m^2^) (**b**) RMSE comparison for different algorithms (RCS = 1 m^2^).

In the second scenario, the RCS value of the targets is increased to 1 m^2^. Detection performances of all algorithms are shown in Figure 9a for different α values. Similar to the results obtained in the first scenario, the detection performance of the proposed method remains superior to the other two methods. In the second scenario, the average PoD value over the entire α range is increased to 0.334 for CAF, 0.742 for FLOSCAF and 0.891 for the proposed algorithm. From the RMSE graph in Figure 9b, it is observed that the average RMSE decreases in a manner similar to that in the first scenario.

**Figure 10:**
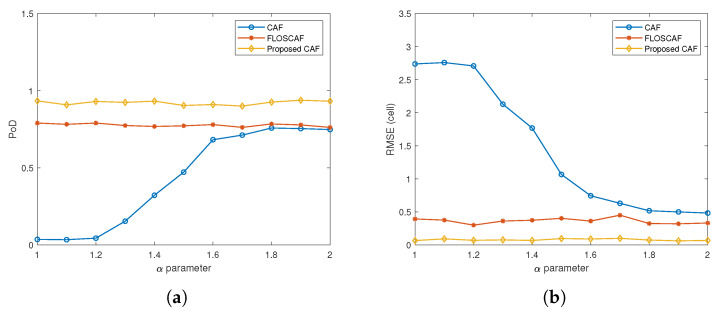
(**a**) PoD comparison for different algorithms (RCS = 2 m^2^) (**b**) RMSE comparison for different algorithms (RCS = 2 m^2^).

Finally, Figure 10 presents the performance comparisons of algorithms for the scenario where the RCS value of the targets is 2 m^2^. As expected, there is considerable difference between the detection performance of the proposed method and than of the other conventional methods. The PoD value of each of the three algorithms increases to 0.428 for CAF, 0.776 for FLOSCAF and 0.921 for the proposed method, while the average RMSE value is reduced in comparison to the other two scenarios in the whole α range.

The above results can be summarized as follows:

- It can be said that the PoD value of the proposed algorithm is higher than these of the other two algorithms throughout the α range.
- While the proposed method and the FLOSCAF method produce consistent results throughout the entire α range, the detection performance of the CAF method increases as the α value increases.
- When RMSE graphs and PoD graphs are considered together, it is observed that the graphs show interrelated results. As the PoD values of the algorithms increase, the RMSE values decrease.
- Finally, it is concluded that as the RCS values of the targets increase, the detection performances of all three algorithms improve.

### 5.2. Experimental Results with Real Data

**Figure 11:**
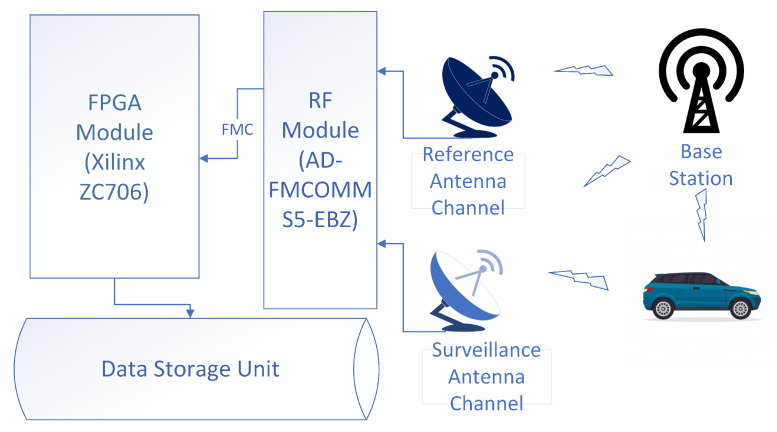
Architecture of the data capture unit.

Real data were collected using a passive radar platform set up by the Estimation, Tracking and Fusion Laboratory at Ankara University. This platform is established by assembling low-cost commercial off-the-shelf (COTS) elements. The measurement platform is composed of antennas and capturing unit. The antenna unit has two parabolic antennas, one of which is positioned directly to receive the reference signal from the UMTS base station while the other collects the reflected signal from the surveillance area, and these antennas are positioned so that the main lobes are least affected from each other. Employed antennas in the system have a gain of 23 dBi in the 1710–2170 MHz band and a narrow beamwidth of 9 degrees. Two important hardware components that comprise the data capturing unit are the FPGA module and the RF module connected to these antennas. The architecture of the data capturing unit is given in Figure 11. The RF module digitizes the signals from the antennas down to the appropriate frequency band. The FPGA module is responsible for converting the data to a specific format and saving them to the data storage unit. The software that provides the communication between the FPGA development kit and the RF card connected to this kit is the last part that completes the data capturing process.

To demonstrate the feasibility of the proposed algorithm in real world problems, real data were recorded using this passive radar platform. The data capturing unit on the measurement platform recorded the reference and surveillance channels with a sampling rate of 10 MHz through a 0.05 s window. The test setup consisted of a single transceiver pair.

#### 5.2.1. Scenarios

To compare the detection performance of the proposed algorithm with the conventional CAF method, we considered three measurement scenarios where differed in terms of bistatic geometry, the target’s RCS and velocity. Ground vehicles cruised on prespecified routes at predetermined velocities to conduct controlled and repeatable experiments, and the measurements of these experiments were collected in the Ankara University Golbasi 50th Year campus.

To obtain the experimental results in Section 5.2.1, the following steps as followed: First, the direct signals on the surveillance signal are removed by preprocessing. In this step, apart from the direct signal cancellation algorithm, no methods such as range compensation algorithms, beam forming techniques are applied to the data. Then, range-Doppler maps are produced by applying the proposed algorithm and CAF to these signals.

**Figure 12:**
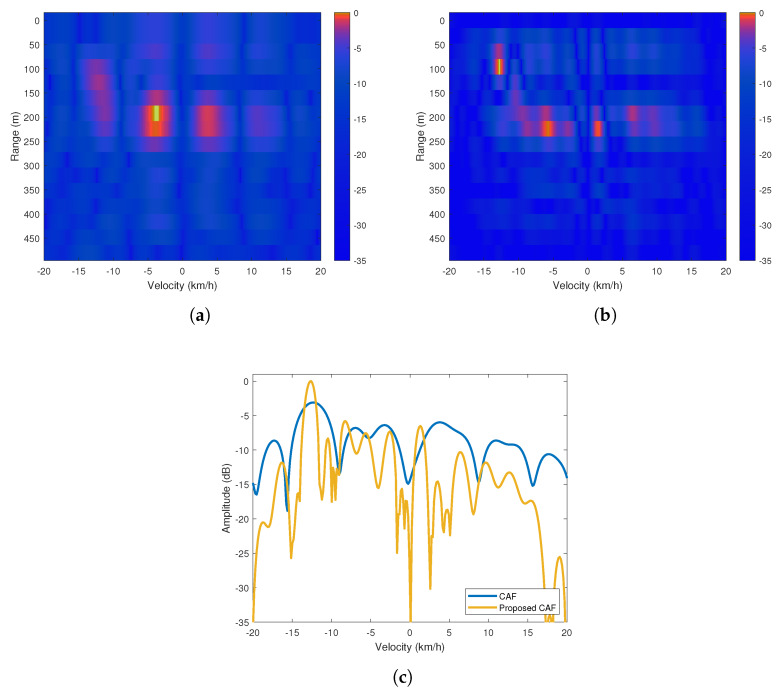
(**a**) Cross Ambiguity Function (CAF) range-Doppler map (**b**) Proposed method’s range-Doppler map (**c**) Doppler profiles of the maps at 75 m.

In the first scenario of the experiments, the vehicle to be detected using the passive radar platform was a mountain bike and the target moved away from the platform at a predetermined constant velocity of approximately 15 km/h at the specified path. The graphs in the first row of Figure 12 are range-Doppler maps generated using the CAF and proposed algorithm. While the expected target appears at 75 meters moving away from the platform at approximately 15 km/h in Figure 12a due to the high performance of the proposed method under impulsive noise, it can hardly be seen in Figure 12b due to the performance degradation of the CAF method under impulsive noise. Since the proposed method suppresses the sidelobes better, the proposed method enhance the visibility of the target on the map. Besides, when the Doppler profiles of the target are compared in Figure 12c, we can see that the maximum power level of the target of interest in the proposed method is 3.1 dB higher than the value obtained in the CAF method. Moreover, the graphs in the first row of Figure 12 show that there exists some strong speckles around 250 m and −5 km/h. These speckles are considered to be an uncontrolled ground vehicle entering the field of view of the measurement platform during the controlled experiment. However, since this target is not involved in the scenario of our controlled experiment, no analysis has been conducted for it.

**Figure 13:**
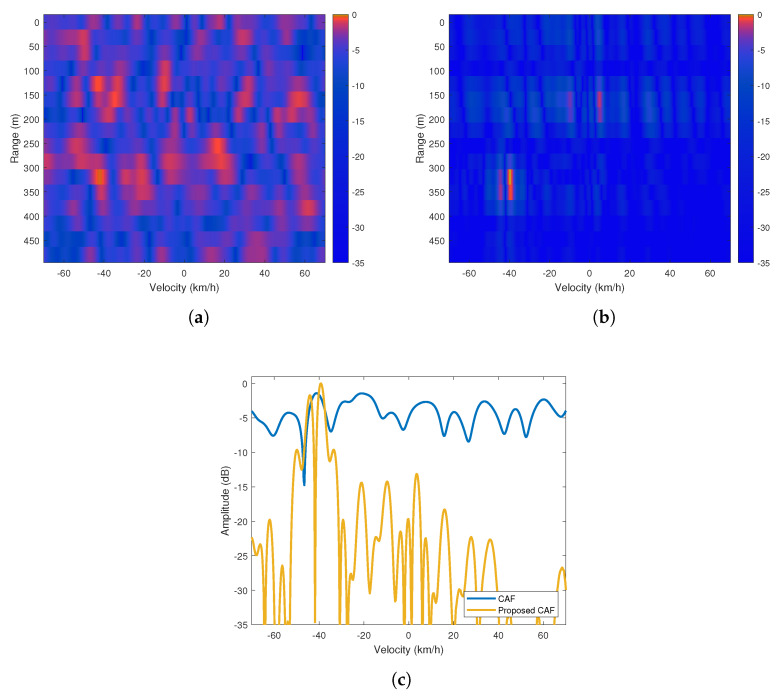
(**a**) CAF range-Doppler map (**b**) Proposed method’s range-Doppler map (**c**) Doppler profiles of the maps at 320 m.

The second scenario has a similar bistatic geometry as the first scenario, but it differs in terms of the RCS of the target. A hatchback model vehicle moved away from the measurement platform at a predetermined constant velocity of 40 km/h. As can be seen in Figure 13a that the target of interest at a constant velocity of 40 km/h on the road is not visible on the CAF range-Doppler map. However, proposed method’s range-Doppler map given in Figure 13b displays a possible detection at the expected range and velocity. In addition, when the Doppler profiles presented in Figure 13c are examined, it is concluded that the target is better distinguished from interference in the proposed method’s range-Doppler map.

**Figure 14:**
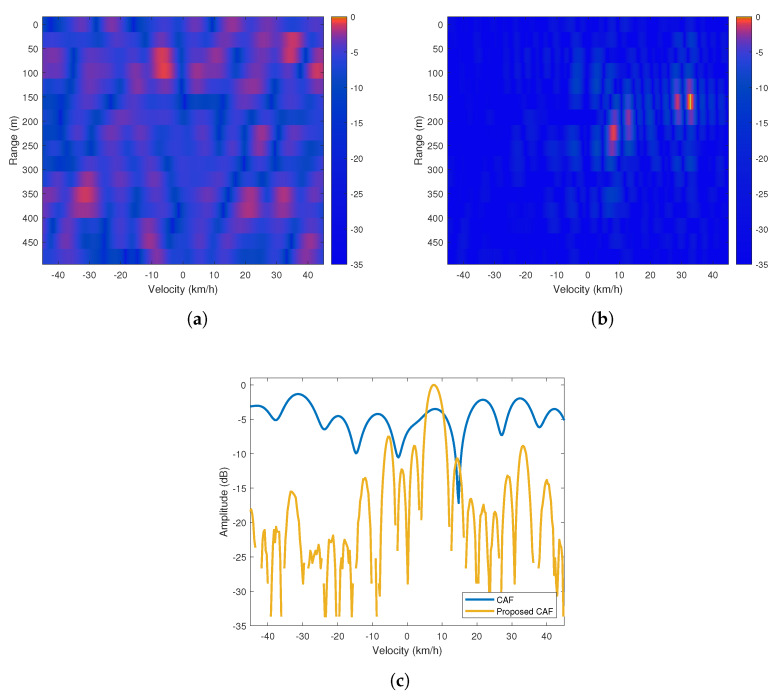
(**a**) CAF range-Doppler map (**b**) Proposed method’s range-Doppler map (**c**) Doppler profiles of the maps at 160 m.

The experiment in the last scenario was carried out by changing the bistatic geometry of the passive radar system using the same vehicle as in the second scenario. The target of interest moved towards the measurement platform at a predetermined constant velocity of approximately 30 km/h. In the last scenario, consistent results are found with the previous scenarios. Figure 14a shows that the target cannot be distinguished due to the interference in the CAF range-Doppler map, whereas, in the proposed method’s range-Doppler map presented in Figure 14b, the target moving towards the platform at a 30 km/h becomes detectable at a distance of 160 meters. Similarly, considering the Doppler profiles of the target at 160 m in Figure 14a, it is observed that there is a difference of 3.5 dB between the target’s maximum energies. Furthermore, when the proposed algorithm and the CAF are compared, it can be concluded that the proposed algorithm provides a better energy concentration since it has higher sidelobe suppression performance and produces a narrower mainlobe than the CAF.

## 6. Conclusions

In this paper, a new algorithm called weighted $l_{1,2}$ CAF was proposed for range-Doppler map generation using a passive radar. The main goal of the new method is to eliminate the performance degradation of conventional methods in the presence of α stable noise. This paper was designed to determine the effect of using the weighted $l_{1,2}$ CAF in the generation of the range-Doppler map under different impulsive noise characteristics. Experimental results on simulated and real data showed that the proposed algorithm is superior to the conventional methods as the characteristic exponent value of the α stable distribution used in the noise model approaches to 1. On the other hand, when the α value approaches 2, the performance of CAF approaches that of the proposed method as expected. However, the proposed method still exhibits lower sidelobe level and higher resolution on the Doppler map than the conventional methods and also provides robust results throughout the range of α values. 

## Figures and Tables

**Table 1:** Time delay and Doppler shift information of the targets in the scenario.

Target	Time Delay Cell	Doppler Shift Cell	Radar Cross Section (RCS) (m^2^)
**1**	2	−6	0.5/1/2
**2**	9	−6	0.5/1/2
**3**	9	−3	0.5/1/2
**4**	12	−1	0.5/1/2
**5**	5	3	0.5/1/2
**6**	10	3	0.5/1/2
**7**	2	6	0.5/1/2
**8**	8	6	0.5/1/2
**A1**	12	0	0.5/1/2
**A2**	2	5	0.5/1/2
